# Highly Variable Bacterial Communities Associated with the Octocoral *Antillogorgia elisabethae*

**DOI:** 10.3390/microorganisms4030023

**Published:** 2016-07-05

**Authors:** Veronica Robertson, Brad Haltli, Erin P. McCauley, David P. Overy, Russell G. Kerr

**Affiliations:** 1 Department of Biomedical Sciences, University of Prince Edward Island, Charlottetown, PE C1A 4P3, Canada; veronica.lynn.kaye@gmail.com (V.R.); bhaltli@upei.ca (B.H.); emccauley@upei.ca (E.P.M.); 2 Department of Chemistry, University of Prince Edward Island, Charlottetown, PE C1A 4P3, Canada; dovery@upei.ca; 3 Department of Pathology and Microbiology, University of Prince Edward Island, Charlottetown, PE C1A 4P3, Canada

**Keywords:** *Pseudopterogorgia elisabethae*, *Antillogorgia elisabethae*, bacterial diversity, microbiome, culture-independent, gorgonian, octocoral, Bahamas, pyrosequencing

## Abstract

*Antillogorgia elisabethae* (synonymous with *Pseudopterogorgia elisabethae*) is a common branching octocoral in Caribbean reef ecosystems. *A. elisabethae* is a rich source of anti-inflammatory diterpenes, thus this octocoral has been the subject of numerous natural product investigations, yet relatively little is known regarding the composition, diversity and the geographic and temporal stability of its microbiome. To characterize the composition, diversity and stability of bacterial communities of Bahamian *A. elisabethae* populations, 17 *A. elisabethae* samples originating from five sites within The Bahamas were characterized by 16S rDNA pyrosequencing. *A. elisabethae* bacterial communities were less diverse and distinct from those of surrounding seawater samples. Analyses of α- and β-diversity revealed that *A. elisabethae* bacterial communities were highly variable between *A. elisabethae* samples from The Bahamas. This contrasts results obtained from a previous study of three specimens collected from Providencia Island, Colombia, which found *A. elisabethae* bacterial communities to be highly structured. Taxa belonging to the *Rhodobacteriales*, *Rhizobiales*, *Flavobacteriales* and *Oceanospiralles* were identified as potential members of the *A. elisabethae* core microbiome.

## 1. Introduction

Branching octocorals, often referred to as gorgonians (Phylum, *Cnidaria*; Order, *Alcyonacea*; Families, *Calcaxonia*, *Holaxonia*, and *Scleraxonia*), are a taxonomically diverse group of sessile marine invertebrates, which are key components of both shallow tropical reefs and deep-sea habitats [1]. In Caribbean waters, gorgonians represent almost 40% of the known invertebrate fauna with over 195 documented species [2]. *Antillogorgia* spp. [3] are the predominant gorgonian octocorals in the Caribbean, with over 15 described species [4]. *Antillogorgia* spp. were previously classified within the genus as *Pseudopterogorgia* prior to a recent revision of gorgonian taxonomy [5]. *A. elisabethae* (syn. *P. elisabethae*) has a moderate distribution in the West Indies and resides in tropical fore reefs at a depth of 5–35 m [4,6]. Morphologically ranging from single plumes to highly branched structures, *A. elisabethae* populations are genetically diverse, exhibiting significant genetic variation within and among populations throughout The Bahamas, Florida, and the San Andres and Providencia Islands of Colombia [6,7,8]. The ability of gorgonians to deter predators via chemical defense may in part explain their dominance on Caribbean reefs [9]. Chemical investigation of gorgonians over the past four decades has uncovered a wealth of structurally diverse bioactive terpene metabolites. *A. elisabethae* is a particularly rich source of diterpenes as 78 compounds, representing 20 distinct terpene skeletons, have been reported from this gorgonian [10]. One important family of diterpenes isolated from *A. elisabethae* is the pseudopterosin family of metabolites, which are used in several marketed cosmetic products for their potent anti-inflammatory properties [11]. Consequently, *A. elisabethae* is unique among octocorals in that wild populations are harvested to supply the commercial demand for pseudopterosins [8]. Due to the added anthropogenic pressure on *A. elisabethae* populations [12] it is important to understand factors affecting the health of this ecologically and economically important octocoral to ensure future viability of wild populations.

Initiated by Rohwer and colleagues in 2001, 16S rDNA-based investigation of coral associated bacterial diversity has revealed that corals contain specific, stable, and diverse symbiotic bacterial communities [13,14,15,16]. In addition to bacteria, coral tissues also host a variety of other microorganisms such as symbiotic dinoflagellates, archaea, fungi, and viruses. The coral “holobiont” is comprised of the coral as well as all of the aforementioned cell types, which live in association with the coral [17]. Although some research suggests cnidarians host species-specific microbial communities [13,14,17], contrasting evidence suggests coral microbiology is more complex than previously assumed. Microbial communities have been shown to differ between conspecifics collected at different sites [18,19,20,21,22], species [23], stages of coral development [24], and spatial location within the holobiont [25]. Despite the ecological importance of octocorals in marine ecosystems and their biotechnological potential as sources of potent bioactive natural products, studies characterizing the microbiomes of octocorals are relatively rare and research has predominantly focused on scleractinian [14,23] and diseased corals [26,27,28]. Studies of octocoral microbial diversity have revealed the presence of diverse bacterial communities, which are distinct from the surrounding environment [18,19,29,30,31,32,33]. The stability of bacterial communities in octocorals appears to vary depending on the gorgonian being investigated. For example, studies of deep-sea octocorals revealed no clear pattern of conserved bacterial consortia [18,19], while the bacterial communities of the Mediterranean octocoral *Paramuricea clavata* were observed to be relatively stable both geographically and temporally [31]. A preliminary study of the bacterial communities associated with three *A. elisabethae* colonies collected from Providencia Island, Colombia uncovered diverse bacterial communities, which were heavily dominated by *Gammaproteobacteria*. At the species level, *A. elisabethae* bacterial communities were predominantly composed of phylotypes related to *Pseudomonas asplenii* and *Endozoicomonas* spp. [34]. In contrast to other octocorals, the bacterial communities of the three individuals examined were highly congruent as six of the ten most abundant phylotypes from each individual were conserved among the three coral individuals. Additionally, these six phylotypes collectively represented a major proportion of the community in each coral specimen (77%–81.2%). This previous study provided an important preliminary assessment of the microbiome of *A. elisabethae*; however, due to the small number of individuals examined, and the limited geographic scope of this study, several questions remain regarding the stability of *A. elisabethae* bacterial communities.

Herein, we employ a 16S rDNA amplicon pyrosequencing approach to describe the bacterial communities of 17 *A. elisabethae* specimens collected from five locations throughout The Bahamas. These communities were also compared to those previously reported from Colombia [34]. The goal of this study was to determine the geographic stability of *A. elisabethae* bacterial communities and to determine if this important octocoral species hosts a stable core microbiome. This research clearly showed that *A. elisabethae* maintains bacterial communities distinct from the surrounding seawater. In contrast to the previous study of Colombian *A. elisabethae* specimens, the microbiomes of Bahamian samples were found to be highly variable between and within sites. Core microbiome analysis indicated that *A. elisabethae* may rely on diverse members of the *Alteromonadales*, *Rhodobacterales*, *Oceanospiralles* and *Flavobacteriales* to fulfill key microbial roles in the holobiont.

## 2. Experimental Section

### 2.1. Sample Collection

*A. elisabethae* octocorals (*n* = 17) were collected in 2006 (June), 2009 (June), 2011 (November) and 2013 (November) by SCUBA from various locations throughout The Bahamas at depths between 15 and 20 m (Table 1; Figure S1). Collection site locations are as follows: Site 1 (Eleuthera), 24 48.55 N, 76 20.58 W; Site 2 (Grand Bahama), 26 36.23 N 77 54.70 W; Site 3a (Bimini), 25 29.13 N, 79 16.41 W; Site 3b (Bimini), 25 31.51 N, 79 17.95 W; Site 4 (Grand Bahama), 26 32.93 N, 78 30.99 W; Site 6a (San Salvador), 24 03.90 N, 74 32.39 W; and Site 6b (San Salvador), 24 03.82 N, 74 32.63 W. Locations and approximate distances between sites (61–1390 km) are provided in Figure S1. For bacterial diversity analyses sites 6a and 6b were treated as a single location due to their close proximity. Coral samples from the same reef were collected ≥10 m apart to reduce the likelihood of sampling clonal propagates and all colonies appeared healthy at the time of collection. Prior to DNA isolation, coral fragments were washed three times by gentle shaking in a tube containing 50 mL of 0.22 µm filtered seawater to remove loosely associated bacteria. Coral samples were frozen on dry ice and stored at −80 °C until DNA was isolated. The identity of all *A. elisabethae* samples were confirmed by extraction of a small clipping and testing for the presence of pseudopterosins, a chemotaxonomic marker useful for the identification of *A. elisabethae* [35]. Seawater in the vicinity of sampled octocorals were collected from sites 3a and 3b (Bimini, 2009) as well as sites 6a and 6b (San Salvador, 2011). Due to technical issues encountered during sampling seawater samples were not collected adjacent to octocorals collected in 2006 and 2013. Seawater (ca. 500 mL) was collected in Ziploc bags and filtered through a 0.22 µm Nalgene MF575 water filtration device (VWR International, Mississauga, ON, Canada). The filter was aseptically removed from the filtration device and frozen at −80 °C until DNA isolation was performed. A negative control Ziploc bag was filled with 500 mL of sterile diH_2_O and filtered as described above to serve as a contamination control.

### 2.2. DNA Isolation

Isolation of holobiont genomic DNA (gDNA) from specimens AE1–AE12 (Table 1) was performed from ~0.5 g of finely chopped octocoral branches using a phenol-chloroform based method as previously described for *A. elisabethae* specimens collected from Colombia [34]. A key difference between this study and the previous report of *A. elisabethae* bacterial diversity was how the octocoral tissue was preserved prior to DNA extraction. In the current study, octocoral samples were frozen on dry ice, while samples from the prior study were stored in phenol [34]. Holobiont gDNA was isolated from specimens AE16–AE20 (~0.25 g of tissue) using the PowerSoil DNA Isolation Kit (Mo Bio Laboratories, Carlsbad, CA, USA) according to the manufacturer’s instructions. In all cases isolated DNA was further purified to remove potential PCR inhibitors using the PowerClean^®^ DNA Clean-Up Kit (Mo Bio Laboratories). To isolate DNA from microbes harvested from the water column, cells were washed off the filter membrane and DNA was extracted and purified using the UltraClean^®^ Water DNA Kit (Mo Bio Laboratories). No 16S rDNA amplicons were obtained from the filtered diH_2_O contamination control. The integrity of purified DNA was assessed by agarose gel electrophoresis and UV absorbance (A_260_/A_280_ ratios varied between 1.73 and 1.95).

### 2.3. Template Preparation for 16S rDNA Amplicon Pyrosequencing

For octocorals collected in 2006 and 2009 (AE1-AE12) nearly full-length small subunit ribosomal RNA (16S) genes amplified from gDNA were used as templates for 16S rDNA pyrosequencing studies. The 16S rRNA genes were amplified using the bacterial universal primers pA (5′-AGAGTTTGATCCTGGCTCAG) and pH (5′-AAGGAGGTGATCCAGCCGCA) [36]. PCR reactions (50 µL) contained 1X EconoTaq Plus Green Master Mix (Lucigen, Middleton, WI, USA), 2.5%–5% molecular biology grade DMSO (Sigma, Oakville, ON, USA), 1 µM each primer and 10–30 ng template DNA. Thermal cycling parameters consisted of 1 cycle at 95 °C for 3 min, 34 cycles of 95 °C for 45 s, 54 °C for 1 min, 72 °C for 1.5 min, and a final extension cycle at 72 °C for 10 min. PCR products were purified using the DNA Clean and Concentrator Kit (Zymo Research, Irvine, CA, USA). Triplicate PCR 16S rDNA amplicons from each coral holobiont gDNA sample were pooled to reduce potential amplification biases [37]. Pyrosequencing was conducted on all other samples using gDNA as a template.

### 2.4. 16S rDNA Amplicon Pyrosequencing 16S rDNA Amplicon Pyrosequencing

Bacterial 16S rDNA amplicons from octocorals and seawater DNA samples collected in 2006 and 2009 were sequenced by Research and Testing Laboratories (RTL; Lubbock, TX, USA) as described previously [38,39]. For octocorals AE1–A12 and seawater samples W1 and W2 a portion of the 16S rRNA gene encompassing the V1 to V3 variable regions was amplified from gDNA samples (W1/W2) or from pooled nearly full-length 16S rDNA PCR amplicons (octocoral samples) (20 ng) using the primer pair pA and 16S519r (5′-GAATTACCGCGGCGGCTG) according to published RTL procedures [39]. Sequences in this dataset spanned the V1 and V2 regions of the 16S rDNA and are herein referred to as the V1/V2 dataset. To assess variability between 16S rDNA pyrosequencing experiments, replicate templates in the form of pooled (triplicate) 16S rDNA amplicons, were generated from the same *A. elisabethae* gDNA sample and sequenced in separate independent sequencing runs (sequence libraries 1A and 1B).

For samples collected in 2011 and 2013 (octocorals AE16–AE20; seawater samples W13–W26), the V4 region of the 16S rRNA gene was subjected to amplicon pyrosequencing using the primer pair F515 (5′-GTGCCAGCMGCCGCGGTAA) and R806 (5′-GGACTACHVGGGTWTCTAAT) and gDNA as template. [40]. These primers were used because they were shown to be more effective at amplifying 16S rRNA gene fragments from small quantities of DNA (personal communication, Dr. Scot Dowd, MR DNA, Shallowater, TX, USA). Sensitivity was an important consideration because we initially attempted to compare the bacterial communities of three tissue fractions from individual octocoral colonies: (1) holobiont (i.e., derived from intact octocoral branches); (2) *A. elisabethae* larvae; and (3) purified zooxanthellae (symbiotic dinoflagellates). Unfortunately, the small quantities of DNA obtained from the larvae and zooxanthellae fractions did not generate sufficient pyrosequencing data to enable a thorough analysis (data not shown). In contrast, DNA isolated from the holobiont tissue samples and seawater samples were not limiting and high quality pyrosequencing results were obtained from these DNA samples (referred to herein as the V4 dataset). While the V4 dataset is not directly comparable to the V1/V2 dataset, it was included in this study to further examine differences between octocoral and water microbiomes. V4 amplicons were generated using HotStarTaq Plus Master Mix Kit (Qiagen, CA, USA) according to the manufacturer’s recommendations. Thermal cycling conditions consisted of 94 °C for 3 min, followed by 28 cycles of 95 °C for 30 s, 53 °C for 40 s and 72 °C for 1 min, followed by a single elongation step at 72 °C for 5 min. Following amplification, the PCR products from different samples were mixed in equal concentrations and purified using Agencourt AMPure beads (Agencourt Bioscience Corporation, MA, USA). Sequencing was performed at MR DNA (Shallowater, TX, USA) utilizing Roche 454 FLX Titanium instruments and reagents according to the manufacturer’s guidelines.

### 2.5. Pyrosequencing Data Analysis

Sequence data for the V1/V2 dataset were processed using MOTHUR version 1.32.0 [41] according to published recommendations [42,43] and as previously described [34]. Briefly, key settings in the data analysis were as follows. The number of flows was trimmed to 450 using trim.flows and data were denoised using PyroNoise (shhh.flows). Sequences were removed if they contained homopolymers greater than 8 bp in length, ambiguous bases, more than two mismatches to the forward primer sequence, more than one mismatch to the barcode sequence or were shorter than 200 bp. Sequences were aligned in MOTHUR using the Silva reference alignment. Alignments were filtered to remove gaps and sequences were preclustered allowing for two nucleotide differences between sequences (diffs = 2). Chimeras were removed using UCHIME. Sequences from three Colombian *A. elisabethae* specimens (SRA043548) [34] were processed with sequences from Bahamian corals collected in this study to ensure consistency in data processing. The V4 dataset was analyzed as described above with a few modifications to accommodate the shorter length of the V4 amplicons. A minimum of 360 flows was set as the minimum number of flows for the trim.flows command and the minimum sequence length was decreased to 150 bp. These quality filtered datasets were used for all subsequent analyses.

### 2.6. Bacterial Community Analysis

To compare the class level community composition between samples, sequences were classified using the MOTHUR Bayesian classifier (80% confidence interval) employing the MOTHUR-formatted version of the Ribosomal Database Project training set (v. 9) [44]. Sequences classified as chloroplasts, mitochondria or “unknown” (i.e., not classified at the domain level) were removed from the analysis. Graphs comparing community composition were prepared using Microsoft Excel 2010. To compare the diversity, membership and structure of bacterial communities from different samples an OTU-based approach was employed. OTUs were identified at the species level using a sequence similarity cutoff of 97% (*D* = 0.03) as described previously [43]. Alpha diversity measures of observed richness (*S*_obs_), and Chao1 estimated richness (*S*_est_), along with the Shannon diversity (*H′*) and equitability (*E*) indices were calculated using MOTHUR [41]. To enable direct comparisons between octocoral samples, these calculations were performed on subsampled datasets (2200 sequences for the V1/V2 dataset; 2600 sequences for the V4 dataset). To compare richness between samples and to evaluate sampling coverage, rarefaction curves were generated using MOTHUR. Statistical comparisons of diversity measures observed between sample groups were performed using MiniTab 17. The Mann–Whitney test was used for pairwise comparisons, while the Kruskal–Wallis test was used to test for differences between three or more groups. To examine beta diversity (similarities or differences between samples) community structure was compared using the Yue and Clayton measure of dissimilarity (θ-YC). Dendrograms displaying the relationships between samples were created from θ-YC distance matrices using the tree.shared command which utilizes the unweighted pair group method with arithmetic mean clustering (UPGMA) algorithm. Trees were viewed using FigTree v. 1.4.0 [45]. To determine if different groups exhibited statistically significant clustering the parsimony and unweighted UniFrac tests were conducted. Similarity percentage (SIMPER) analysis was utilized to calculate similarities between bacterial communities of samples collected from a single site, as well as to calculate dissimilarities between sites. Analyses were conducted on percent normalized data matrices of class level taxa distribution among samples. SIMPER analysis was conducted using PRIMER v. 5.2.4 (Primer-E Ltd., Lutton, UK) [46]. Putative members of the core microbiome were defined as species level OTUs observed in ≥50% of *A. elisabethae* samples [47]. OTUs were excluded from this list if their average abundance in seawater samples was greater than in *A. elisabethae* samples. Principle component analysis (PCA) was used to model the distribution of octocoral-associated bacterial diversity by geographic location in the V1/V2 dataset. PCA was conducted using the species level, percent normalized, OTU data matrix (OTUs defined as variables). PCA was performed in Unscrambler X (version 10.1; Camo ASA, Oslo, Norway) using the single value decomposition algorithm and validated by cross validation.

### 2.7. Accession Numbers

Pyrosequencing data have been archived in the NCBI Sequence Read Archive (SRA) under Bioproject accession number PRJNA313050. The alpha numeric identifier of a representative sequence for each specific OTU referred to in the results is provided in Table S1. These sequences can be retrieved from the raw data contained in the SRA.

## 3. Results

### 3.1. Bacterial Community Analysis

After stringent quality filtering using MOTHUR to reduce the effects of PCR and sequencing artifacts, 98,273 sequences were obtained from 17 *A. elisabethae* specimens collected from five locations throughout The Bahamas. Seven seawater samples yielded 43,282 sequences (Table 1). Inclusion of sequences obtained from a previous study of bacterial diversity in three specimens of *A. elisabethae* from Colombia [34] contributed an additional 12,935 sequences. The average sequencing depth (±s.d.) obtained for octocorals in the V1/V2 and V4 datasets were 5516.1 ± 1804.8 and 4590.2 ± 1268.7, respectively. Similar sequencing depth was obtained for seawater samples (V1/V2—4721.5 ± 1572.5, V4—6359 ± 2374.6). Sequencing depths were not significantly different between groups defined on the basis of 16S region sequenced, sample type, and collection site (Kruskal–Wallis test; P = 0.464) (Table 1). Rarefaction curves were prepared to estimate sampling coverage (Figure S2). The curves did not approach the asymptote for the majority of samples, indicating that further sampling depth would recover additional species level (*D* = 0.03) diversity.

The richness and diversity observed in *A. elisabethae* bacterial communities ranged widely between octocoral samples, particularly within the V1/V2 dataset. After subsampling to reduce the effect of unequal sequencing depths (V1/V2, *n* = 2200; V4, *n* = 2600) average observed richness (±s.d.) in the V1/V2 and V4 datasets were 209.19 ± 133.81 and 132.20 ± 35.71, respectively. Similar variability (as indicated by relatively large standard deviations) was observed for estimated richness (V1/V2—502.25 ± 313.88 OTUs; V4—281.20 ± 105.07 OTUs), the Shannon diversity index (V1/V2—2.61 ± 1.15; V4—2.61 ± 0.41) and the Shannon evenness index (V1/V2—0.50 ± 0.16; V4—0.54 ± 0.07). No statistically significant differences in alpha diversity statistics (*S*_obs_, *S*_est_, *H′* and *E*) were observed between groups defined on the basis of sampling site (V1/V2 sites), collection year (San Salvador 2011 vs. 2013) or 16S rDNA region analyzed (V1/V2 vs. V4 dataset).

To determine if the observed wide range in diversity measures between individual octocorals was due to experimental variation inherent in the sequencing methodology we compared diversity estimates from replicate sequence libraries 1A and 1B, which were independently generated from the same gDNA sample. Observed richness was identical between the replicate libraries and the other diversity measures were very similar, exhibiting coefficients of variation (CVs) ranging between 2.5% and 5.5%. This level of variation is far less than that observed among octocoral samples in the V1/V2 dataset where CVs ranged from 33% for *E* to 64% for *S_obs_*.

To compare bacterial diversity between seawater and octocoral samples, W13–W26 were compared to H17, H18 and H26, as these samples were collected in parallel in San Salvador. Observed richness, and Shannon diversity and evenness was significantly greater in seawater samples (Mann–Whitney tests; *P* = 0.0369 for each test) (Table S2).

**Figure 1:**
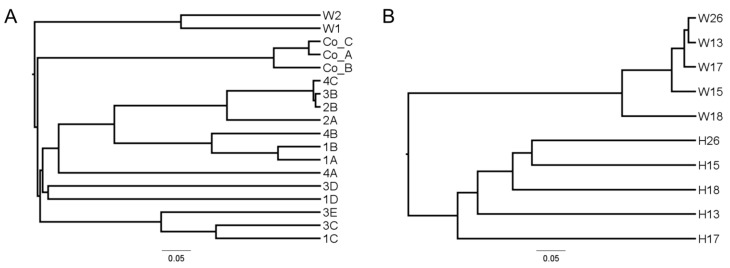
Comparison of microbial communities from *A. elisabethae* and seawater using the Yue and Clayton dissimilarity calculator: (**A**) V1/V2 dataset subsampled to 2200 sequences/library; and (**B**) V4 dataset subsampled to 2600 sequences/library. Dendrograms were prepared using the UPGMA algorithm.

An OTU-based approach was used to compare the structure of octocoral and seawater bacterial communities at the species level (*D =* 0.03) (Figure 1). The V1/V2 and V4 datasets were analyzed separately, as OTUs derived from different regions are not directly comparable [48]. In cluster analyses based on the Yue and Clayton theta dissimilarity measure seawater samples clustered apart from octocoral samples in both datasets (Figure 1A,B). This clustering was significant as parsimony and unweighted UniFrac tests returned P values less than 0.05 (Table S3), indicating that the structure of octocoral bacterial communities were significantly different than those of the seawater samples.

In the V1/V2 dataset, Colombian samples formed a distinct clade from the Bahamian octocorals (Figure 1A), which was supported by parsimony and unweighted UniFrac tests (*P* < 0.017; Bonferroni corrected significance level for a three way comparison between seawater, Bahamian octocorals and Colombian octocorals) (Table S3). The short branch lengths in Figure 1A indicated that the bacterial communities of Colombian samples were highly similar. No consistent clustering by site was observed among octocorals collected from Bahamian sites 1–4 (Figure 1A). Replicate sequence libraries 1A and 1B clustered tightly together in Figure 1A, illustrating the similarity of these libraries. In the V4 dataset, which consisted of octocorals collected in two different years from sites separated by approximately 500 m no apparent clustering by site or year was apparent (Figure 1B).

**Figure 2:**
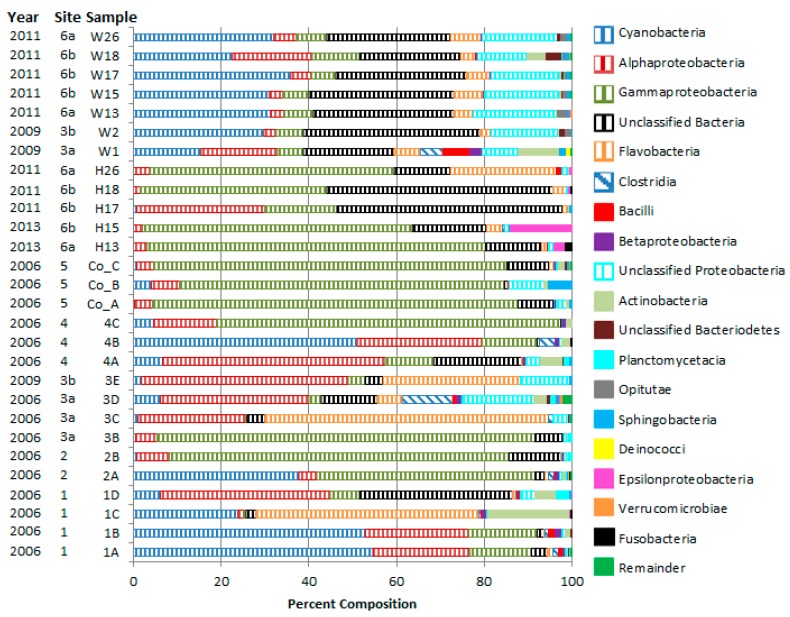
Class level bacterial community composition of *A. elisabethae* and seawater samples. Collection year and site are indicated adjacent to sample identifier. “Unclassified Bacteria” refers to sequences that could not be classified by the RDP classifier using a confidence threshold of 80%. “Remainder” is an artificial category encompassing classes that represented <1% of sequences in all sequence libraries. This category accounts for 0.0%–2.21% of each bacterial community.

Sequences derived from all *A. elisabethae* samples analyzed in this study (*n* = 20) could be assigned to 15 phyla and 25 recognized classes while those from the seven seawater samples could be assigned to 14 phyla and 21 classes (Figure 2 and Tables S4 and S5). In general, the most abundant members (±s.d.) of *A. elisabethae* bacterial communities were *Gammaproteobacteria* (40.5% ± 32.5%), *Alphaproteobacteria* (16.9% ± 15.7%, Unclassified Bacteria (12.8% ± 14.9%), *Cyanobacteria* (12.2% ± 18.8%), and *Flavobacteria* (9.0% ± 17.8%) (Figure 2, Table S4). The abundance of these groups varied considerably between octocorals collected both from the same site and from different sites (Figure 2). Similarity percentage analysis (SIMPER) of class level bacterial communities revealed variable intra-site similarity between bacterial communities of octocorals collected from Bahamian sites 1–4 (1—42.8%, 2—58.8%, 3a—23.07%, and 4—38.9%). Greater overall intra-site similarity was observed in octocoral samples from San Salvador (59.1% similarity) and Colombia (86.4% similarity). Average inter-site dissimilarity (±s.d.) between 2006 Bahamian sites was 62.4% ± 8.9%. Similar levels of dissimilarity were observed between 2006 Bahamian sites and the Colombian site (60.4% ± 17.9%) and between 2006 Bahamian sites and San Salvador (63.4% ± 12.3%). Dissimilarity between the Colombian site and San Salvador was 38.5%. We again assessed internal experimental variability by comparing the taxonomic composition derived from replicate libraries 1A and 1B. SIMPER analysis revealed a low degree of dissimilarity (5.9%) between the class level taxonomic composition of these samples.

Seawater bacterial communities were primarily composed of *Cyanobacteria*, Unclassified Bacteria, and Unclassified *Proteobacteria*, which collectively accounted for 44.3%–85.4% of sequences in seawater samples (Table S5). SIMPER analysis revealed considerable dissimilarity between comparable seawater and *A. elisabethae* bacterial communities (3E vs. W1/W2—65.8%; H17–H26 vs. W12–W26—60.47%). Taxa contributing to the dissimilarity between seawater and octocoral samples are summarized in Table S5.

### 3.2. Core Microbiome of *A. elisabethae*

**Figure 3:**
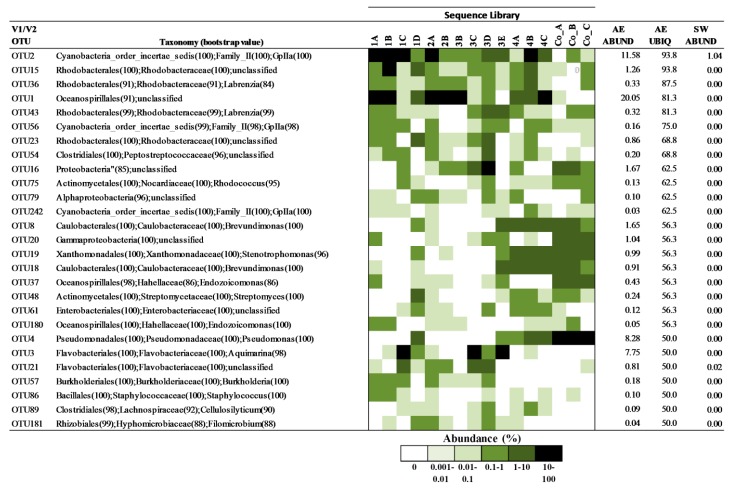
Heat map showing the distribution of OTUs in the V1/V2 dataset comprising the putative core microbiome of *A. elisabethae* in 16 sequence libraries prepared from 15 *A. elisabethae* colonies (1A and 1B are replicate libraries prepared from the same colony). Taxonomic classification determined by the RDP Classifier is shown. AE ABUND—average OTU abundance (%) across all *A. elisabethae* sequence libraries. AE UBIQ—percentage of *A. elisabethae* sequence libraries in which OTU was detected. SW ABUND—average OTU abundance (%) in seawater samples W1 and W2.

**Figure 4:**
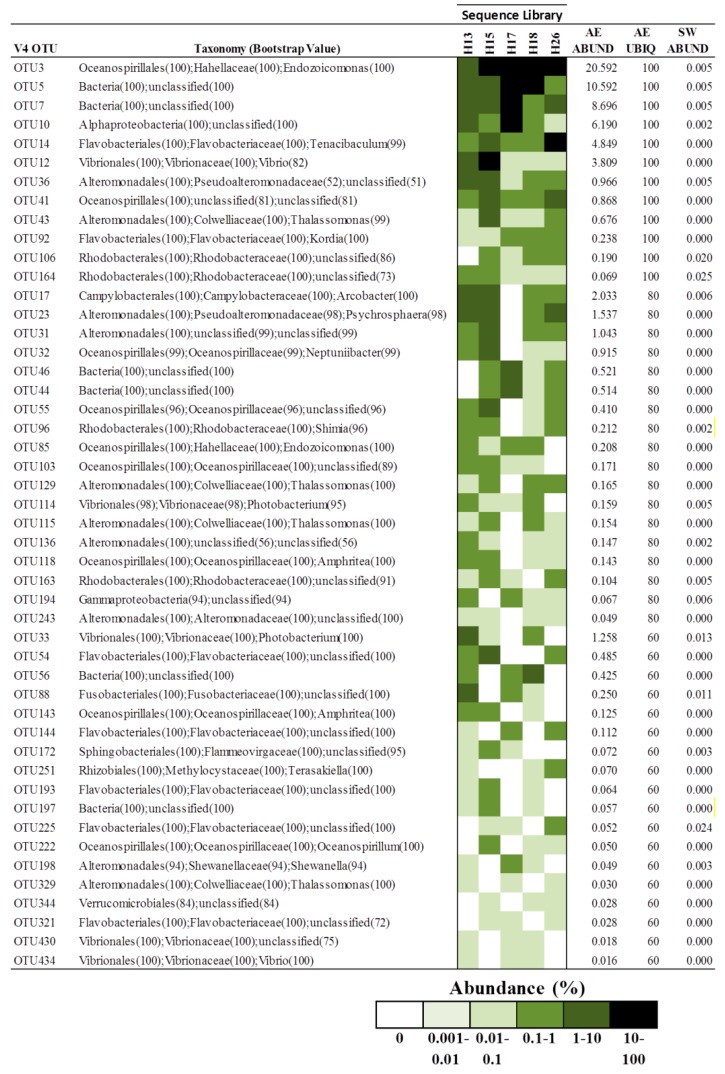
Heat map showing the distribution of OTUs in the V4 dataset comprising the putative core microbiome of five *A. elisabethae* colonies. Taxonomic classification determined by the RDP Classifier is shown. AE ABUND—average OTU abundance (%) across all *A. elisabethae* sequence libraries. AE UBIQ—percentage of *A. elisabethae* sequence libraries in which OTU was detected. SW ABUND—average OTU abundance (%) in seawater samples W13–W26.

Previous analyses of marine invertebrate microbiomes have shown that many organisms maintain a core set of bacterial associates that are stably maintained geographically and temporally, irrespective of changing environmental conditions [20,30]. To identify putative members of the *A. elisabethae* microbiome we identified OTUs that were present in >50% of octocoral samples at any abundance. OTUs were excluded from the list if they were found in greater average abundance in seawater samples than in octocoral samples, as these OTUs may not represent true octocoral bacterial associates but rather carry over from the surrounding seawater. The distribution of OTUs fulfilling these criteria in the V1/V2 and V4 datasets are shown in Figure 3 and Figure 4, respectively. In the V1/V2 dataset 27 OTUs were identified as candidate members of the core microbiome (Figure 3). *Alphaproteobacteria* and *Gammaproteobacteria* constituted the greatest proportion of the putative core microbiome (29.6% and 22.2%, respectively). The average (±s.d) and median abundance of putative core microbiome OTUs were 2.2% ± 4.5% and 0.33%, respectively. Interestingly, six OTUs previously identified as abundant in Colombian samples were included in the current list of putative core microbiome members. These OTUs were classified as *Brevundimonas* sp. (OTUs 18 and 8), *Stenotrophomonas* sp. (OTU19), *Pseudomonas* sp. (OTU4), *Endozoicomonas* sp. (OTU37) and Unclassified *Gammaproteobacteria* (OTU20). Five OTUs were encountered in >75% of *A. elisabethae* samples. OTUs 15, 36 and 43 were *Rhodobacteraceae*, and had average abundances between 0.32% and 1.26%. OTUs 1 and 2 exhibited high average abundances (>10%) and were observed in a high proportion of *A. elisabethae* samples (>80%). OTU1 had the greatest average abundance (20.05% ± 30.1%) and was observed in all Bahamian octocorals collected in 2006 and one of three Colombian samples, but was absent from 3E and the seawater samples. This OTU was identified as an unidentified *Oceanospiralles* by the RDP classifier with 97% confidence. BlastN analysis revealed OTU1 showed strong sequence identity (98.2%–100%) to uncultured bacteria detected in the octocorals *Eunicella cavolini* (Mediterranean Sea) [30], *Eunicella singularis* (Mediterranean Sea), *Eunicella verrucosa* (SW coast of England) [33] and *Gorgonia ventalina* (Caribbean Sea, Panama) [49]. OTU2 was assigned to the GPIIa group of *Cyanobacteria* and BlastN analysis indicated it was closely related to several *Synechococcus* spp. (100% identity). OTU2 had a high average abundance in octocorals (11.58% ± 16.9%) and was absent from only one octocoral (Co_A). This OTU was also found in both seawater samples, albeit at a significantly lower average abundance (1.04% ± 0.58%).

In the V4 dataset, forty-eight OTUs met the criteria to be considered a component of the putative core microbiome (Figure 4). *Gammaproteobacteria* (56.3%) and *Alphaproteobacteria* (10.4%) comprised the majority of OTUs; however OTUs associated with *Flavobacteria* were more prevalent (16.7%) in the V4 core microbiome. At the genus level, OTUs classified as *Alteromonadales*, *Flavobacteriales* and *Oceanospiralles* were the most commonly encountered taxa, accounting for 14.6%–22.9% of putative core microbiome OTUs. *Vibrionales* (8.3%) and *Rhodobacteriales* (10.4%) also accounted for a significant portion of OTUs in Figure 4. The average (±s.d) and median abundance of putative core microbiome OTUs was 1.45% ± 3.5% and 0.18%, respectively. Twelve OTUs (25%) were present in all octocoral samples, with average abundances ranging from 20.6% (OTU3) to 0.069% (OTU164). Six of these OTUs (3, 5, 7, 10, 12 and 14) were found in all octocorals with high average abundances (3.8%–20.6%). OTU12 and 14 were classified as *Vibrio* and *Tenacibaculum* species, respectively. OTU10 was an unclassified *Proteobacteria* that showed limited similarity to any cultured bacterium but 97.5% similarity to a clone from the gorgonian *Corallium rubrum* (KP008700.1). OTUs 5 and 7 could not be classified below the domain level by the RDP classifier and showed low identity (<85%) with any cultured bacterium. The most similar sequences in GenBank to OTU5 were to two 16S rDNA clones from the Alaskan octocoral *Cryogorgia koolsae* (93.3%–93.5% identity) (HM173225.1, HM173211.1). OTU7 exhibited high similarity (98.0% identity) to 46 16S rDNA clones from the octocoral *C. rubrum* collected from the Mediterranean Sea (e.g., KP008776.1). OTU3 was assigned to the genus *Endozoicomonas* by the RDP classifier with high confidence and showed 99.5% sequence identity to *E. euniceicola* EF212^T^, previously isolated from the octocoral *Eunicea fusca* [50].

### 3.3. Characterizing Dominant Bacteria Driving the Separation of Coral-Associated Bacterial Communities by PCA

**Figure 5:**
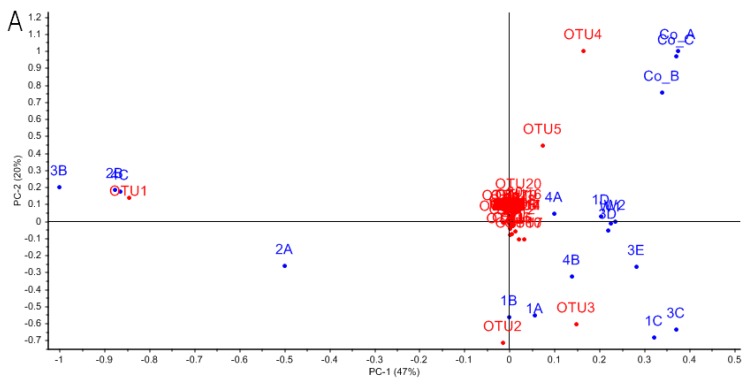

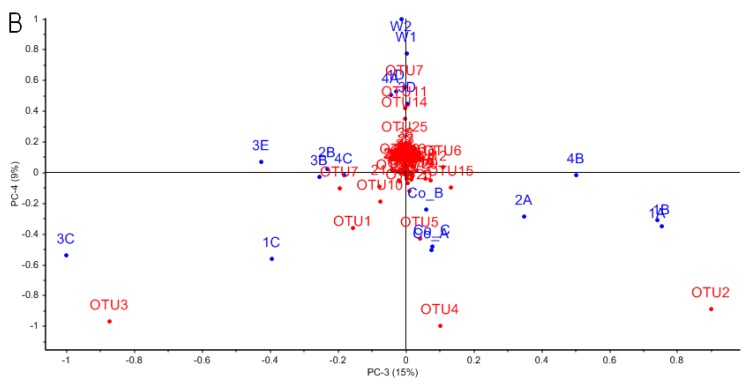
PCA biplots describing species level OTUs responsible for the observed variance between *A. elisabethae* and seawater bacterial communities. Red circles labeled with “OTU” numbers represent OTUs. Blue circles represent *A. elisabethae* and seawater bacterial communities. PC1 and −2 (**A**) describe 50% and 19% of the observed variance, respectively. PC3 and −4 (**B**) describe 15% and 8% of the variance, respectively. Collectively PC1–4 explains 92% of the total variance.

The lack of consistent structure in *A. elisabethae* bacterial communities in the V1/V2 dataset cluster analysis prompted us to utilize PCA (Figure 5) to identify phylotypes driving the dissimilarity between bacterial communities in Figure 2A. An explained variance plot (Figure S3) indicated that the model optimized after 4 PCs, explaining 92% of the model variance. In the biplot of PC1 and PC2 (Figure 5A) negative separation along PC1 was driven by OTU1 (unclassified *Oceanospiralles*), which distinguishes the bacterial communities of *A. elisabethae* samples 2A, 2B, 3B and 4C. OTU1 was detected in 13 of 16 samples but was highly represented in these five sequence libraries (Figure 3). Negative separation of samples along PC2 was driven by OTU2 and OTU3. Separation of samples 1A, 1B, 1C, 2A and 4B along PC2 was driven by OTU2, which was particularly abundant in these samples (34.8% ± 11.0%). OTU2 (*Synechococcus* sp.) was present in all other octocoral samples (except Co_A) and the two seawater samples; however abundance in these samples was comparatively low (1.0% ± 1.4%) (Figure 3). OTU3 drove separation of 1C, 3C and 3E in a positive direction along PC1 and represented a major component of the bacterial community of these samples (41.1.1% ± 13.2%) (Figure 3). OTU3 was assigned to the genus *Aquimarina* by the RDP classifier (99% confidence) and exhibited 94.5% sequence identity with *A. penaei* isolated from the gut of the Pacific white shrimp [51]. Positive separation along PC2 was driven by OTUs that were abundant in Colombian octocorals (OTUs 4, and 5) but only sporadically encountered at low frequencies in Bahamian octocoral samples (Figure 3). These OTUs corresponded to *Pseudomonas* (OTU4)- and *Endozoicomonas* (OTU5)-related OTUs previously reported as major constituents of the microbiomes of *A. elisabethae* samples collected from Colombia [34].

PC3 and PC4 (Figure 5B) modeled differentiation of bacterial communities due to the influences of OTUs 1 through 5, with similar trends to those described for PC1 and PC2. Differentiation between octocoral and seawater samples was modeled by positive separation along PC4, primarily driven by OTUs 7, 11, 14 and 25 (Figure 5B). These phylotypes could be assigned to genera typically associated with seawater such as phototrophic GPIIa *Cyanobacteria* (OTU7) and oligotrophic “*Candidatus* Pelagibacter” (OTUs 11, 14 and 25) [49,52]. These OTUs accounted for 3.4%–22.3% of sequences in seawater samples, but were absent or present at very low abundances (≤0.6%) in octocoral samples (data not shown).

## 4. Discussion

*A. elisabethae* is widely distributed throughout the Caribbean and is of particular biotechnological and commercial interest because it is the sole source of the pseudopterosin family of anti-inflammatory diterpenes [11]. An initial study of the bacterial diversity associated with three *A. elisabethae* specimens collected from the far western range of this octocoral (Providencia Island, Colombia), indicated that the bacterial communities were highly structured with the majority (77%–81%) of the bacterial communities belonging to six OTUs affiliated with the genera *Pseudomonas* and *Endozoicomonas* [34]. In the current study, we expanded the analysis of the *A. elisabethae* microbiome to include an additional 17 coral specimens collected from five sites in The Bahamas. Samples from the surrounding seawater were also obtained to determine if *A. elisabethae* hosts bacterial communities distinct from its surroundings. This analysis allowed us to further expand our knowledge base regarding the microbiome of *A. elisabethae* and assess the geographic stability of bacterial communities between sites separated by <1 km to >1400 km (Figure S1). We also examined the temporal stability of *A. elisabethae* bacterial communities collected from San Salvador where replicate samples were obtained in two different years.

Whether invertebrates’ host specific microbial communities are distinct from their surroundings is a fundamental question in the study of marine invertebrate microbiomes. To answer this question we compared the microbiomes of seven seawater samples to 17 *A. elisabethae* samples. Seawater samples were collected in three different years from two sites (Bimini and San Salvador) separated by approximately 500 km and analyzed using different 16S hypervariable regions. In all analyses, seawater samples were more diverse and clearly differentiated from those of *A. elisabethae* at both the class and species levels. PCA analysis identified OTUs classified as GPIIa *Cyanobacteria* and “*Candidatus* Prochlorococcus” as being responsible for the majority of the variance between octocoral and seawater samples in the V1/V2 dataset. The prevalence of these oligotrophic bacteria in seawater is consistent with other studies of seawater microbial communities [49,53,54,55,56]. One seawater-associated OTU (OTU2, V1/V2 dataset), identified as a *Synechococcus* sp., was ubiquitous and abundant in octocoral samples. *Synechococcus* spp. are common seawater inhabitants which are ingested by corals, so it is possible that *A. elisabethae* may simply be ingesting these bacteria as a food source and their presence in the microbiome may reflect a partially digested meal [57]. It is also possible that the relationship is more complex as *Synechococcus* spp. have been shown to contribute to nitrogen fixation in the scleractinian coral *Montastreae cavernonsa* [58] and have been implicated in disease outbreaks in the Mediterranean octocorals *E. cavolini* and *E. singularis* [59]. Additional research is required to further elucidate the role of *Synechococcus* in *A. elisabethae*. Despite the abundance of *Synechococcus* in several *A. elisabethae* samples, our data clearly demonstrate that *A. elisabethae* hosts specific bacterial communities, distinct from the surrounding seawater.

Examination of 17 *A. elisabethae* specimens in this study revealed a lack of consistent species level community structure between octocorals collected at the same site/year, as well as between those collected from different sites/years. Bacterial diversity also ranged widely between octocoral samples. This is in contrast to a previous study of the microbiome of Colombian *A. elisabethae*, which exhibited highly similar bacterial communities [34]. In the current study, the bacterial communities of the Colombian samples clustered apart from Bahamian samples in cluster and PCA analyses. This result is not surprising as they were collected >1400 km from the closest Bahamian site and in a different year, thus temporal and/or geographic factors may be responsible for the observed differences in bacterial community composition and structure. Unlike the Colombian samples, the bacterial communities of *A. elisabethae* from different sites in the V1/V2 dataset did not exhibit high intra-site similarity as indicated by low SIMPER similarity scores. In fact, the average inter-site similarity between Bahamian sites was comparable to intra-site similarity levels. Cluster analysis of the V1/V2 OTU dataset also showed no apparent location-based clustering of octocorals from The Bahamas as has been reported for other corals [22,60]. These results suggest that the composition and structure of *A. elisabethae* bacterial communities varies as much at local geographic scales as it does over much larger regional distances. The bacterial communities of octocorals collected from San Salvador (V4 dataset), which were collected in two different years, did not cluster according to collection year, suggesting that a consistent shift in the microbiome of *A. elisabethae* did not occur at these two time points.

Variability in bacterial diversity and community structure between specimens has previously been reported for several other octocorals [19,30,32,33], as well as scleractinian corals [60]. Significant fluctuation in the diversity and structure of coral bacterial communities indicates that these bacterial assemblages are highly dynamic. Studies of other octocorals have linked microbiome variations to depth, salinity, habitat disturbance, proximity to preferred habitats and coral health [19,20,30,32]. All *A. elisabethae* samples were collected within a relatively narrow depth range (15–20 m) and in the open ocean, thus it is unlikely that depth or salinity were key drivers of variability in *A. elisabethae*, although we cannot rule out the influence of salinity as we did not measure this parameter. Disease is not likely responsible for variations in *A. elisabethae* microbiome structure and diversity as all colonies appeared healthy at the time of collection; however, the presence of undetected underlying disease cannot be ruled out. Anthropogenic disturbance is also an unlikely factor, as significant variations in bacterial diversity and community structure were observed between colonies collected from the same site (*~*20–40 m apart), which would likely experience similar levels of disturbance from sources such as pollution. If anthropogenic disturbances are responsible for shifts in microbiome structure and diversity, the disturbances must occur on a highly localized scale and may be caused by insults to individual octocoral colonies (e.g., interactions with divers or with fishing gear). The recent proposition by Roder et al. [20] suggesting that the bacterial diversity present in *Ctenactis echinata* correlates with proximity to preferred habitats is intriguing in respect to *A. elisabethae*. We did not assess the population density of *A. elisabethae* at the collection sites used in this study; however, the abundance of this octocoral is highly variable through its range and can vary drastically between adjacent, yet seemingly similar habitats [8]. It would be interesting to determine if bacterial diversity and community structure correlate with habitat suitability for *A. elisabethae*. *A. elisabethae* exhibits a high-degree of morphological variation, as well as variation in natural product content and composition [6,61], indicating substantial physical and biochemical plasticity between coral colonies, which could affect microbiome diversity and structure. This is particularly true in regard to diterpene content as several pseudopterosin congeners have reported antimicrobial activity and could mediate microbiome dynamics [62,63].

Methodological variation has been recognized as a source of variation in previous microbiome pyrosequencing studies [64,65] and could be responsible for a portion of the variation observed in Bahamian *A. elisabethae* bacterial communities. We assessed variability inherent in our sequencing methodology by analyzing replicate sequence libraries 1A and 1B, which exhibited very similar diversity and community structure. While not a thorough analysis of methodological variation, these data suggest that intrinsic variation in amplicon generation and pyrosequencing methodology were not major drivers of observed variation between samples. Differences in DNA isolation efficiency and resulting purity of DNA are additional factors that can affect the results of amplicon pyrosequencing studies [65]. Isolation of *A. elisabethae* holobiont gDNA that was a suitable template for PCR was challenging; use of the PowerClean^®^ Clean-Up Kit was essential to generating PCR compatible DNA, suggesting that PCR inhibitors were frequently co-extracted with *A. elisabethae* DNA. DNA was assessed for quality by electrophoresis and absorbance (A_260_/A_280_), however, these techniques would leave many potential contaminants undetected. *A. elisabethae* tissues contain high concentrations of natural products [10], thus it is possible that some of these compounds (or others) will be co-extracted with DNA and affect downstream processes.

Collectively, the data presented here suggest that *A. elisabethae* colonies from The Bahamas host highly variable bacterial assemblages. This observation presents challenges to studying factors that influence microbial diversity and community structure in *A. elisabethae* as these data suggest that highly localized factors may significantly influence the microbiome of this octocoral. Future analyses should consider the effect of environmental parameters (at local and regional scales), population density and *A. elisabethae* morphological and chemical characteristics on microbiome structure and diversity. Methodology should also be carefully considered for future studies to ensure true variations in diversity can reliably be differentiated from methodological noise. Stringent standardization of methodology and analysis of multiple biological replicates from individual octocoral colonies should help quantify and mitigate the effect of methodological variation [64].

Previous culture-independent examinations of octocoral bacterial diversity from deep and shallow water environments have established *Gammaproteobacteria* and *Alphaproteobacteria* as major members of octocoral microbial communities [18,19,30,31,32,49,66]. The contribution of other taxa to octocoral microbiomes appears to be variable and may be dependent on environmental conditions, coral species and health status of the host octocoral. In general, *Gammaproteobacteria* and *Alphaproteobacteria* were the dominant taxa in *A. elisabethae*, with high-levels of *Cyanobacteria*, *Flavobacteria* and unclassified Bacteria occurring sporadically, typically at the expense of *Gammaproteobacteria* abundance. The association of *Gammaproteobacteria* and *Alphaproteobacteria* with *A. elisabethae* appears to be robust as a similar trend was observed in analyses of both V1/V2 and V4 regions. This association also appears to be temporally stable as corals collected from San Salvador Island in 2011 and 2013 show similar class level taxonomic composition.

While *Gammaproteobacteria* and *Alphaproteobacteria* appeared to be stably associated with *A. elisabethae* specimens distributed throughout The Bahamas, we sought to identify bacterial phylotypes at a lower taxonomic level that may play a key role in the *A. elisabethae* holobiont. Consequently, we attempted to identify the core microbiome of *A. elisabethae* using an inclusive approach, which identified putative members of the core microbiome as species level OTUs, which were present in a minimum of 50% of octocoral samples, with no minimum abundance criteria. Recent research by Hester and colleagues indicates that relatively rare OTUs often exhibit a greater distribution frequency (ubiquity) among coral samples than would be expected based on their abundance and thus should not be discounted on the basis low abundance. These phylotypes are considered “stable” coral symbionts. Conversely, highly abundant OTUs with lower than expected ubiquity may be considered “sporadic” symbionts [67]. Previous research identified six OTUs that were both highly abundant and ubiquitous in three *A. elisabethae* samples from Colombia [34]. These OTUs were also detected in Bahamian samples but were sporadically distributed among Bahamian *A. elisabethae* samples at low abundances, suggesting that these phylotypes are likely sporadic symbionts which are widely distributed in the *A. elisabethae* population. Due to the non-overlapping nature of the V1/V2 and V4 datasets direct comparison of OTUs from the two datasets was not possible, thus two putative core microbiomes were assembled. Notable taxa present in the V1/V2 putative core microbiome which were absent in the V4 counterpart were *Brevundimonas*, GPIIa *Cyanobacteria*, *Pseudomonas* and *Stenotrophomonas*. Conversely, OTUs belonging to the *Vibrionales* and *Alteromonadales* were frequently encountered in the V4 core microbiome but absent in the V1/V2 list. The fact that these taxa are found only one core microbiome list may suggest that these OTUs are sporadic octocoral symbionts. Amplification biases associated with the different hypervariable regions may also have prevented equal detection of these taxa in both datasets [65].

Only four taxa were present in both the V1/V2 and V4 core microbiomes: *Oceanospiralles*, *Rhodobacterales*, *Rhizobiales* and *Flavobacteriales*. The detection of these taxa in both datasets suggests they may have a stable association with *A. elisabethae* and play important roles in the holobiont. Multiple *Oceanospiralles* associated OTUs were present in both core microbiomes and one OTU from each dataset was both abundant and ubiquitous (V1/V2 OTU1; V4, OTU3). The *Oceanospiralles* OTUs could be assigned to the genera *Endozoicomonas*, *Amphritea* and *Oceanospirillum*, while several could not be classified below the order level. Members of the *Oceanospiralles* are aerobic heterotrophs that have been identified in other octocorals [30,33,49,50] and scleractinian corals [15,24,49,60], as well as a wide variety of marine invertebrates including sponges [68,69], molluscs [70], ascidians [71], polychaetes [72], starfish [73] and sea anemones [74]. It has been proposed that members of the *Oceanospiralles* have developed evolutionarily old symbiotic associations with octocorals [30]. Due to the widespread abundance of *Oceanospiralles* in octocorals it is likely that they play an important ecological role. Currently, little is known about the functions played by *Oceanospiralles* in the holobiont. Members of this order have been shown to metabolize dimethylsulphoniopropionate (DMSP), suggesting a role in sulphur cycling in scleractinian corals [75]. Dinoflagellate symbionts (*Symbiodinium* sp.), which are believed to be the source of DMSP in scleractinian corals, are also present in *A. elisabethae* [75,76]. Thus it is possible that *Oceanospiralles* are involved in sulphur cycling in this octocoral. Reduction of nitrate to nitrite has also been observed for members of the *Oceanospiralles*, thus these microbes may also play a role in nitrogen cycling in the holobiont [50]. *Oceanospiralles* are also known for their ability to produce hydrolytic enzymes, which can assist their host in acquiring nutrients in otherwise oligotrophic environments [72]. In addition, these abundant symbionts may also play a probiotic role in *A. elisabethae* through the production of antibiotics [77] or through competitive exclusion of pathogens [78]. To elucidate the role of *Oceanospiralles* in octocorals, further effort must be focused on culturing these important symbiotic bacteria so that their metabolic capabilities and genetic potential can be directly analyzed.

*Rhizobiales* and *Rhodobacterales* are *Alphaproteobacteria* which are known for their diverse metabolisms. One OTU belonging to the *Rhizobiales* was detected in each of the core microbiomes. *Rhizobiales* are known diazotrophs and have been implicated in the nitrogen cycle in *Acropora millepora* [79]. Members of this group may also be involved in sulphur cycling as some members of this group have been shown to degrade methanesulphonate [80]. *Rhodobacterales* were the second group of *Alphaproteobacteria* present in both core microbiomes. *Rhodobacterales* are ubiquitous in marine environments and have been detected in seawater as well as corals such as *Lophelia purtusa* [81], *C. echinata* [20] and the octocoral *E. singularis* [82]. The potential functions of *Rhodobacterales* symbionts may include the production of antimicrobial agents, as well as nutrient cycling [83]. *Rhizobiales* and *Rhodobacteriales* affiliated OTUs in the core microbiome list were typically present at low abundances in *A. elisabethae* samples but with a high ubiquity, suggesting these phylotypes may represent stable symbionts.

*Flavobacteriales* were present in the V1/V2 and V4 core microbiomes. In both cases one *Flavobacteria*-affiliated phylotype was found at a high abundance in a few samples (V1/V2 OTU3; V4 OTU 14), suggesting these phylotypes are sporadic symbionts. *Flavobacteriales* have been implicated in diseases of several corals such as white band disease in *Acropora cervicornis* [26,84]. *Flavobacteriales* are typically aerobic, heterotrophic, gliding bacteria which produce flexirubin-type pigments and are widely distributed in marine environments. Genomic evidence suggests that *Flavobacteriales* are involved in carbon cycling in marine environments through the degradation of biopolymers such as chitin. Genes for a complete denitrification pathway as well as sulphate reduction have been found in *Flavobacteriales* genomes suggesting these organisms are involved in nitrogen and sulphur cycling [85]. *Flavobacteriales* may also help mediate biofouling through the production of algicidal substances [86].

The putative members of the *A. elisabethae* core microbiome described above have been implicated in a wide array of processes which may provide benefit to the holobiont as a whole. Future research employing metagenomics and transcriptomics will be required to determine the extent to which bacterial symbionts contribute to the overall metabolism of the holobiont and to link those activities to specific bacterial phylotypes.

## 5. Conclusions

We have presented the first in-depth study on the geographic and temporal variability of bacterial communities in the gorgonian octocoral *A. elisabethae* and have demonstrated that this coral maintains bacterial communities distinct from the surrounding seawater. The bacterial communities of *A. elisabethae* were found to be highly variable within The Bahamas. This high level of variability was apparent in terms of overall diversity (i.e., richness and evenness) as well as taxonomic diversity. This contradicts an initial study of three *A. elisabethae* specimens originating from Providencia Island, Colombia, which showed *A. elisabethae* maintained highly similar bacterial communities [34]. The discrepancy between these studies is likely due to the small sample size in the earlier study, although careful consideration should be paid to methodological standardization in future studies to reduce the potential effects of methodological variation. Attempts to identify a core microbiome in *A. elisabethae* identified 75 potential members. Four taxa represented in the list of candidate microbiome members were found to consistently associate with *A. elisabethae*. *Oceanospiralles* in particular appear to have a persistent association with *A. elisabethae* and may perform important ecological functions in the holobiont. Future studies should attempt to identify biotic and abiotic factors that drive variations in the *A. elisabethae* microbiome. In light of the high variability between bacterial communities of *A. elisabethae* colonies collected from the same site, special care will need to be paid to sampling strategies to ensure that fine scale variations in environmental parameters are not overlooked and to ensure that the effects of methodological variations on microbiome analyses can be accounted for. In summary, the research presented here has significantly expanded our knowledge of the diversity and stability of bacterial communities associated with *A. elisabethae*. This research has set the stage for further studies to determine factors that drive microbiome dynamics in this ecologically and economically important octocoral.

## Figures and Tables

**Table 1:** Sample collection/processing information and alpha diversity analysis of *A. elisabethae* (AE) and seawater (W) samples. DNA isolation methods: PC—phenol-chloroform, UW—UltraClean^®^ Water DNA Kit, PS—PowerSoil DNA Isolation Kit. DNA template type used for pyrosequencing analysis: PCR—nearly full-length 16S rDNA PCR amplicons, gDNA—genomic DNA. Alpha diversity statistics: *S*_obs_—observed richness, *S*_est_—estimated richness (Chao1 index), *H′*—Shannon diversity index, *E*-Shannon equitability index. All calculations were conducted on OTU level data.

Sequence Library	Site	Sample	Year	DNA Isol. Meth.	Pyroseq. Template	16S rDNA Region	No. Reads	Avg. Length (bp)	*S*_obs_^a^	*S*_est_^a^	*H′* ^a^	*E* ^a^
1A ^b^	1	AE1	2006	PC	PCR	V1/V2	4685	222	253	514	2.47	0.44
									*162*	*385*	*2.43*	*0.48*
1B ^b^	1	AE1	2006	PC	PCR	V1/V2	4969	222	260	444	2.25	0.41
									*162*	*366*	*2.21*	*0.43*
1C	1	AE2	2006	PC	PCR	V1/V2	6962	240	162	216	2.01	0.40
									*90*	*179*	*1.98*	*0.44*
1D	1	AE3	2006	PC	PCR	V1/V2	5221	228	886	1978	5.13	0.79
									*524*	*1295*	*5.05*	*0.81*
2A	2	AE4	2006	PC	PCR	V1/V2	7200	233	310	533	1.90	0.37
									*151*	*379*	*1.85*	*0.41*
2B	2	AE5	2006	PC	PCR	V1/V2	8884	245	404	785	1.71	0.28
									*181*	*414*	*1.64*	*0.32*
3B	3a	AE6	2006	PC	PCR	V1/V2	2424	245	182	471	1.20	0.23
									*170*	*442*	*1.19*	*0.23*
3C	3a	AE7	2006	PC	PCR	V1/V2	5906	243	366	1032	1.99	0.34
									*184*	*563*	*1.93*	*0.37*
3D	3a	AE8	2006	PC	PCR	V1/V2	2754	235	565	1245	4.77	0.75
									*497*	*1054*	*4.73*	*0.76*
3E	3b	AE9	2009	PC	PCR	V1/V2	7038	228	530	1434	3.52	0.56
									*248*	*726*	*3.44*	*0.62*
4A	4	AE10	2006	PC	PCR	V1/V2	5560	231	598	1290	4.48	0.70
									*356*	*768*	*4.38*	*0.75*
4B	4	AE11	2006	PC	PCR	V1/V2	6450	221	233	556	2.74	0.50
									*128*	*280*	*2.71*	*0.56*
4C	4	AE12	2006	PC	PCR	V1/V2	7269	241	144	338	1.30	0.26
									*72*	*165*	*1.27*	*0.30*
Co_A ^c^	5	AE13	2010	PC	gDNA	V1/V2	5831	247	152	333	2.03	0.40
									*85*	*210*	*2.00*	*0.45*
Co_B ^c^	5	AE14	2010	PC	gDNA	V1/V2	2258	248	91	197	2.41	0.54
									*90*	*195*	*2.41*	*0.54*
Co_C ^c^	5	AE15	2010	PC	gDNA	V1/V2	4846	244	411	842	2.58	0.43
									*247*	*615*	*2.52*	*0.46*
W1	3a	W1	2009	UW	gDNA	V1/V2	3149	232	155	206	3.86	0.76
									*140*	*184*	*3.85*	*0.78*
W2	3b	W2	2009	UW	gDNA	V1/V2	6294	223	325	563	3.25	0.56
									*195*	*366*	*3.19*	*0.61*
H13	6a	AE16	2013	PS	gDNA	V4	3759	201	219	457	3.10	0.58
									*178*	*395*	*3.08*	*0.59*
H15	6b	AE17	2013	PS	gDNA	V4	5511	201	164	314	3.10	0.61
									*116*	*219*	*3.10*	*0.65*
H17	6b	AE18	2011	PS	gDNA	V4	6247	201	147	351	2.12	0.42
									*94*	*180*	*2.10*	*0.46*
H18	6b	AE19	2011	PS	gDNA	V4	4775	201	249	514	2.57	0.47
									*172*	*422*	*2.54*	*0.49*
H26	6a	AE20	2011	PS	gDNA	V4	2659	201	102	192	2.25	0.49
									*101*	*190*	*2.25*	*0.49*
W13	6a	W3	2011	UW	gDNA	V4	3341	201	216	300	3.35	0.62
									*197*	*276*	*3.33*	*0.63*
W15	6b	W4	2011	UW	gDNA	V4	4278	201	235	358	3.34	0.61
									*194*	*296*	*3.32*	*0.60*
W17	6b	W5	2011	UW	gDNA	V4	7706	201	352	518	3.27	0.57
									*213*	*331*	*3.22*	*0.60*
W18	6b	W6	2011	UW	gDNA	V4	7979	201	409	695	3.80	0.63
									*254*	*414*	*3.74*	*0.68*
W26	6a	W7	2011	UW	gDNA	V4	8491	201	317	526	3.36	0.58
									*195*	*324*	*3.31*	*0.63*

^a^ Italicized numbers calculated using subsampled datasets. Sequence libraries covering the V4 region were subsampled to 2600 sequences while those covering the V1/V2 region were subsampled to 2200 sequences; ^b^ Replicate sequence libraries prepared from the same genomic DNA sample; ^c^ Data from previously reported study [34].

## Supplementary Materials

The following are available online at www.mdpi.com/2076-2607/4/3/23/s1.
