# Supplementary material for: Highly Variable Bacterial Communities Associated with the Octocoral Antillogorgia elisabethae
Source: Microorganisms. 2016 Jul 5;4(3):23. doi: 10.3390/microorganisms4030023 (PMC5039583; doi:10.3390/microorganisms4030023)
Supplement: Supplementary file 1 [file microorganisms-04-00023-s001.pdf]

microorganisms

MDPI

Supplementary Materials: Highly Variable Bacterial Communities Associated with the Octocoral *Antillogorgia elisabethae*

Veronica Robertson [1,†], Brad Haltli [1,2,†], Erin P. McCauley [1], David P. Overy [2,3] and Russell G. Kerr [1,2,*]

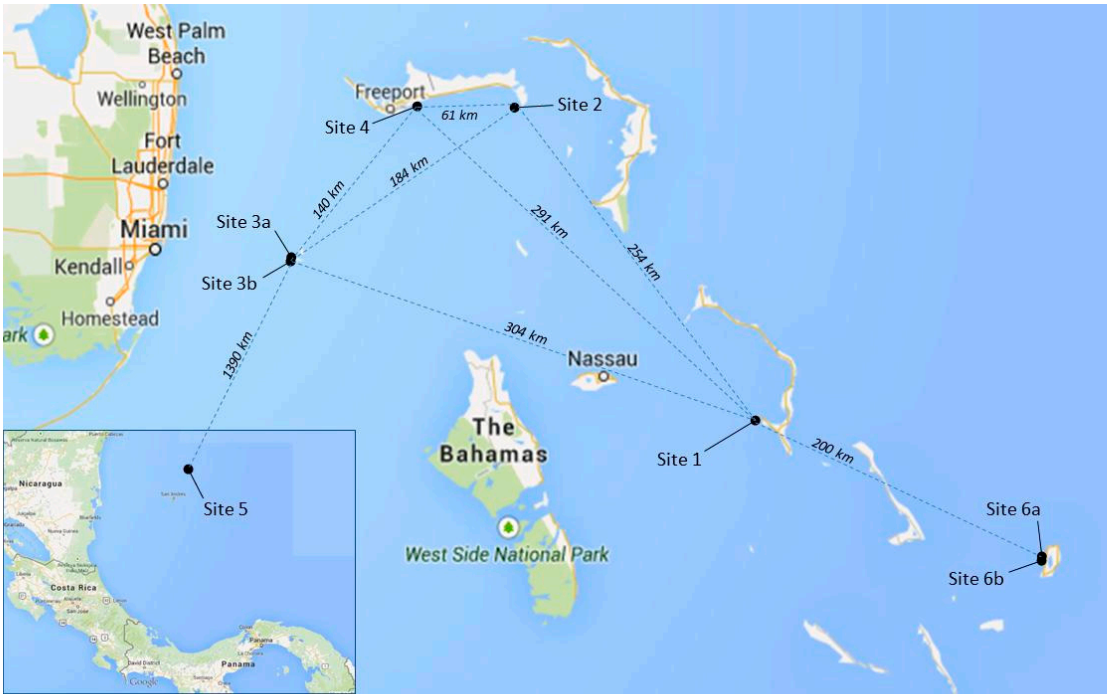

Figure S1. *A. elisabethae* sampling locations. Inset map shows the location of Providencia Island, approximately 1390 km from Bimini, The Bahamas. Approximate distance between collection sites is indicated adjacent to blue dashed lines. Map prepared using Google Maps™ mapping service. Google and the Google logo are registered trademarks of Google Inc., used with permission.

A **B**

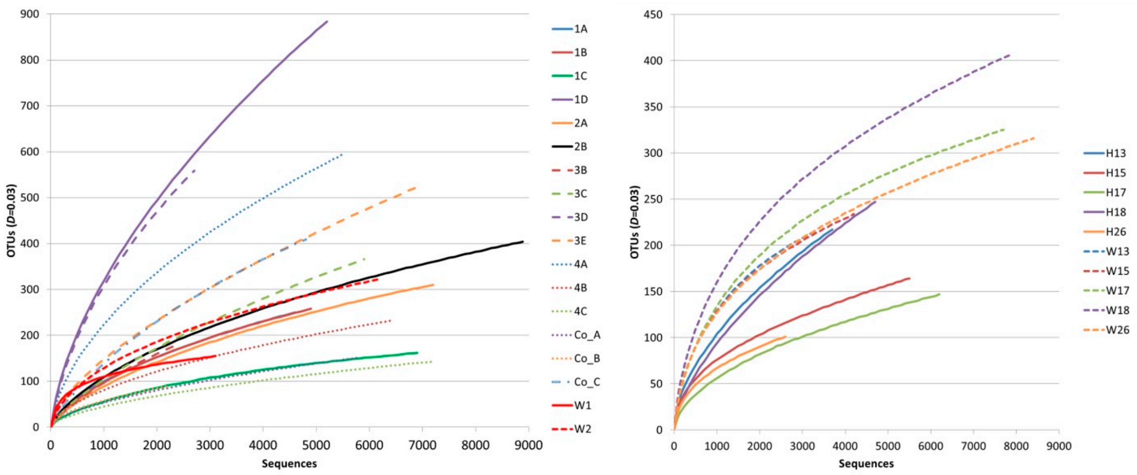

Figure S2. Rarefaction curves for *A. elisabethae* and seawater 16S rDNA libraries at 3% dissimilarity. A, rarefaction analysis of 16S rDNA sequences libraries covering the V1 and V2 regions. B, rarefaction analysis of 16S rDNA sequences libraries covering the V4 region.

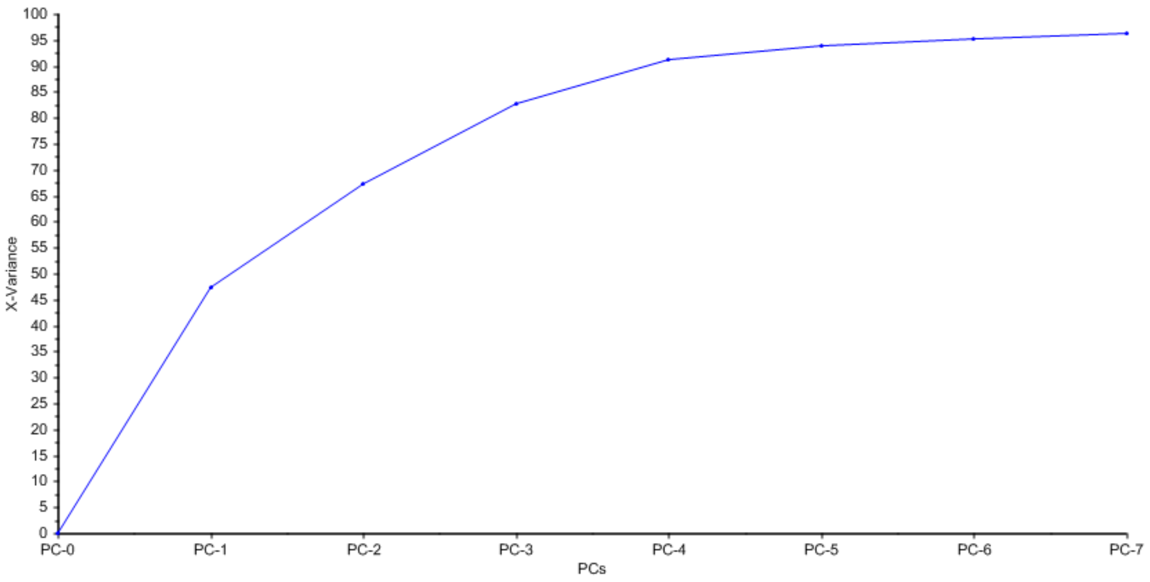

Figure S3. Explained variance plot showing explained (x)-variance reaches an asymptote at 4 PCs.

Table S1. Alpha numeric code for representative sequences (Rep. Seq.) for each OTU mentioned in the results. Sequences can be retrieved from the NCBI Sequence Read Archive (SRA) under Bioproject accession number PRJNA313050.

**V4 Dataset OTUs**		**V1/V2 Dataset OTUs**	
**OTU**	**Rep. Seq**	**OTU**	**Rep. Seq.**
OTU0003	H8Y4T4E02IY3WH	OTU0001	GF13U7302DWR0W
OTU0005	H8Y4T4E02GUN56	OTU0002	GF13U7302DB06L
OTU0007	H8Y4T4E02JSI5B	OTU0003	GF13U7302D0DJV
OTU0010	HXC1SGT02I303H	OTU0004	GYBHR2K02C6LQW
OTU0012	H8Y4T4E02INYCB	OTU0008	GF13U7302E10SR
OTU0014	HXC1SGT02IK70	OTU0015	GF13U7302DVIP6
OTU0017	H8Y4T4E02IV1W3	OTU0016	GF13U7302EEV5G
OTU0023	HXC1SGT02IMA6V	OTU0018	GYBHR2K02ES9BF
OTU0031	H8Y4T4E02HDUP1	OTU0019	GF13U7302DEEPU
OTU0032	H8Y4T4E02H48TI	OTU0020	GF13U7302ELB6C
OTU0033	HXV11MC01C55OH	OTU0021	GF13U7302DDSYD
OTU0036	H8Y4T4E02HTA8H	OTU0023	GF13U7302DWT0U
OTU0041	H8Y4T4E02H8HVX	OTU0036	GYBHR2K02EQY10
OTU0043	H8Y4T4E02FYX5A	OTU0037	GYBHR2K02DJFS5
OTU0044	H8Y4T4E02GS8E9	OTU0043	GF13U7302ETX6W
OTU0046	H8Y4T4E02HF6HS	OTU0048	GYBHR2K02DSKC9
OTU0054	H8Y4T4E02HBE4Z	OTU0054	GF13U7302C2ZL6
OTU0055	H8Y4T4E02IMUNU	OTU0056	GYBHR2K02EWFPD
OTU0056	HXC1SGT02FW6E4	OTU0057	GF13U7302DRR8E
OTU0085	H8Y4T4E02HOGM4	OTU0061	GF13U7302C9HYN
OTU0088	H8Y4T4E02H0TK5	OTU0075	GF13U7302ESYU7
OTU0092	H8Y4T4E02HCA5W	OTU0079	GF13U7302D619L
OTU0096	H8Y4T4E02JEY7V	OTU0086	GF13U7302DW9VO
OTU0103	HXV11MC01B898D	OTU0089	GF13U7302EUQOB
OTU0106	HXC1SGT02G5SDE	OTU0180	GF13U7302DG9KV
OTU0114	HXC1SGT02HAFLR	OTU0181	GF13U7302EKRFN
OTU0115	HXV11MC01DFROB	OTU0242	GF13U7302DYTYZ
OTU0118	HXV11MC01DRHL1		
OTU0129	HXV11MC01AYLTC		
OTU0136	HXV11MC01C4I3Q		
OTU0143	HXV11MC01EMF8M		
OTU0144	HXV11MC01C41BD		

Table S1. *Cont.*

OTU0163	HXV11MC01DDL87
OTU0164	H8Y4T4E02HTBIG
OTU0172	HXV11MC01DA7FW
OTU0193	HXV11MC01CQRF0
OTU0194	H8Y4T4E02G8MOG
OTU0197	HXV11MC01BYHTR
OTU0198	H8Y4T4E02JFPNY
OTU0222	H8Y4T4E02H6ATR
OTU0225	H8Y4T4E02IMLE4
OTU0243	HXV11MC01AXZ3H
OTU0251	H8Y4T4E02J4ZHC
OTU0321	H8Y4T4E02GCAF2
OTU0329	HXV11MC01DWF91
OTU0344	H8Y4T4E02I04PM
OTU0430	HXV11MC01ACPEJ
OTU0434	H8Y4T4E02JA7IJ

Table S2. Statistical comparison (Mann-Whitney test) of bacterial diversity between coral (H17, H18, H26) and seawater samples (W13–W26) collected from San Salvador. Abbreviations: Shannon diversity (*H′*) and evenness (*E*), observed (S_{obs}) and estimated (S_{est}) richness. Tests were conducted in MiniTab 17 and differences were considered significant if $P < 0.05$.

Diversity Measure	Sample	Median	P
S_{obs}	coral	101	0.0369
	water	197	
S_{est}	coral	190	0.5510
	water	324	
H′	coral	2.25	0.0369
	water	3.32	
E	coral	0.49	0.0369
	water	0.63	

Table S3. Statistical significance (P) of tests used to compare the community structure of (1) bacterial communities in *A. elisabethae* and seawater and (2) the structure of bacterial communities from *A. elisabethae* collected from different geographic locations. BS—The Bahamas, CO—Colombia, UW—unweighted.

		Yue-Clayton Cladogram	
**Comparison**	**Data Set**	**Parsimony**	**UW-UniFrac**
Octocoral vs. Seawater [a]	V1/V2	0.031	0.002
BS vs. CO Octocorals [b]	V1/V2	0.004	0.011
Octocoral vs. Seawater [a]	V4	0.004	0.007

[a] Significance level P = 0.050; [b] Bonferoni-corrected significance level *IP* = 0.017.

Table S4. Class level community composition (%) of *A. elisabethae* bacterial communities. Abbreviations: nd—not detected, tr—trace abundance (<0.1%).

Class	1A	1B	1C	1D	2A	2B	3B	3C	3D	3E	4A	4B	4C	Co_A	Co_B	Co_C	H13	H15	H17	H18	H26
Acidobacteria	nd	nd	tr	tr	nd	nd	nd	nd	tr	nd	tr	tr	nd	nd	nd	nd	nd	nd	nd	nd	nd
Actinobacteria	0.4	1.1	18.5	4.8	0.7	0.2	tr	0.3	3.0	0.1	5.1	2.1	1.3	0.7	1.1	1.4	0.2	tr	0.1	tr	0.2
Alphaproteobacteria	21.9	23.7	1.1	38.8	3.9	7.5	4.8	24.6	33.7	47.1	50.7	28.6	14.2	4.1	6.4	3.8	3.1	2.0	29.3	1.3	3.9
Bacilli	1.1	1.5	0.3	0.5	0.2	tr	nd	nd	0.8	tr	0.3	0.1	tr	nd	tr	tr	tr	tr	nd	nd	1.1
Bacteroidia	tr	nd	tr	tr	tr	tr	nd	0.1	tr	tr	0.1	tr	tr	tr	tr	nd	nd	nd	nd	tr	nd
Betaproteobacteria	0.4	1.3	1.0	0.2	1.1	0.2	0.2	tr	1.3	tr	0.3	1.0	0.6	0.2	tr	0.1	tr	tr	nd	nd	tr
Chlamydiae	nd	nd	nd	nd	nd	nd	nd	nd	nd	nd	nd	nd	nd	nd	nd	nd	tr	nd	nd	0.1	nd
Clostridia	1.2	1.0	0.3	0.3	1.3	0.2	0.1	1.2	11.7	tr	0.2	3.4	0.2	0.2	tr	0.2	tr	0.6	tr	0.1	tr
Cyanobacteria	54.6	52.7	23.9	6.2	37.8	0.8	0.7	1.1	6.2	2.0	6.7	51.0	4.8	0.3	4.3	0.9	tr	tr	0.8	0.3	tr
Deinococci	nd	nd	nd	tr	nd	nd	nd	nd	nd	nd	nd	nd	nd	nd	nd	nd	nd	nd	nd	nd	nd
Deltaproteobacteria	tr	tr	nd	tr	tr	nd	tr	0.1	0.9	tr	nd	nd	nd	nd	nd	0.2	nd	nd	nd	tr	tr
Epsilonproteobacteria	nd	nd	tr	tr	nd	nd	nd	nd	tr	nd	nd	nd	nd	nd	nd	nd	2.3	13.8	nd	0.2	0.2
Epsilonproteobacteria	nd	nd	tr	tr	nd	nd	nd	nd	tr	nd	nd	nd	nd	nd	nd	nd	nd	nd	nd	nd	nd
Erysipelotrichia	tr	tr	nd	nd	tr	nd	nd	nd	0.9	nd	nd	nd	nd	nd	nd	nd	nd	nd	nd	nd	nd
Flavobacteria	1.4	tr	50.6	0.6	0.9	0.3	tr	64.3	5.6	31.3	0.1	tr	nd	tr	nd	1.0	1.3	3.5	1.1	3.1	24.2
Fusobacteria	0.1	nd	0.1	nd	0.4	tr	nd	tr	nd	tr	nd	0.2	tr	tr	nd	tr	1.5	nd	0.2	tr	nd
Gammaproteobacteria	14.0	15.6	0.7	6.7	49.7	77.3	86.0	0.3	2.8	3.8	11.2	12.3	78.4	83.3	73.9	80.4	77.1	61.6	16.3	42.7	55.5
Gemmatimonadetes	nd	nd	nd	nd	nd	nd	nd	nd	nd	nd	nd	nd	nd	nd	nd	nd	nd	nd	nd	nd	nd
Lentisphaeria	nd	nd	nd	nd	nd	nd	nd	nd	nd	nd	nd	nd	nd	tr	nd	tr	nd	nd	nd	tr	nd
Mollicutes	tr	nd	nd	nd	nd	nd	nd	nd	nd	nd	nd	nd	nd	nd	nd	nd	nd	nd	nd	nd	nd
Negativicutes	nd	nd	nd	nd	nd	nd	nd	nd	nd	nd	nd	tr	nd	nd	nd	nd	nd	nd	nd	nd	nd
Opitutae	tr	nd	nd	tr	tr	nd	nd	nd	0.5	nd	nd	tr	nd	nd	nd	0.3	nd	nd	tr	nd	nd
Planctomycetacia	0.2	0.1	tr	3.3	0.6	0.7	0.9	0.3	1.3	tr	1.8	nd	tr	nd	nd	0.1	nd	tr	tr	nd	0.2
Sphingobacteria	nd	tr	tr	tr	tr	nd	nd	tr	0.4	0.3	tr	nd	nd	0.4	5.2	0.3	tr	0.3	0.3	tr	nd
Spirochaetes	nd	tr	tr	nd	nd	tr	nd	nd	nd	nd	nd	nd	nd	nd	nd	nd	nd	nd	nd	nd	nd
Verrucomicrobiae	nd	nd	nd	tr	tr	nd	nd	tr	0.4	nd	nd	nd	nd	tr	tr	tr	nd	tr	tr	0.1	tr
**TM7 *incertae sedis***	nd	nd	nd	nd	nd	nd	nd	nd	nd	nd	nd	nd	nd	nd	nd	nd	nd	nd	nd	nd	nd
**Unclassified Bacteria**	3.5	1.6	2.3	34.8	2.3	11.8	6.3	4.0	12.9	3.9	19.9	0.6	0.4	8.1	0.8	9.8	12.8	16.8	51.5	51.3	12.7
**Unclassified *Bacteriodetes***	0.2	0.2	0.3	tr	tr	tr	nd	0.2	0.9	tr	0.2	tr	tr	0.2	0.1	0.4	nd	0.1	tr	0.3	nd
**Unclassified *Chloroflexi***	nd	nd	nd	nd	nd	0.1	tr	nd	nd	nd	nd	nd	nd	nd	nd	nd	nd	nd	nd	nd	nd
**Unclassified *Cyanobacteria***	tr	nd	tr	tr	tr	nd	nd	nd	nd	nd	nd	nd	nd	nd	tr	0.1	nd	tr	nd	nd	nd
**Unclassified *Firmicutes***	tr	nd	nd	nd	tr	nd	nd	nd	tr	nd	tr	nd	nd	nd	nd	nd	nd	nd	nd	nd	nd
**Unclassified *Planctomycetes***	nd	nd	nd	tr	nd	nd	nd	nd	0.2	nd	nd	nd	nd	nd	nd	nd	nd	nd	nd	nd	nd
**Unclassified *Proteobacteria***	0.4	1.0	0.4	3.5	0.8	0.8	0.8	3.2	16.3	11.2	3.3	0.5	tr	2.3	8.0	0.7	1.3	1.1	0.2	0.3	1.7
**Unclassified *Verrucomicrobia***	nd	nd	nd	nd	tr	nd	nd	tr	nd	nd	nd	nd	nd	nd	nd	tr	nd	nd	nd	tr	nd
***Verrucomicrobia* Subdivision3**	nd	nd	nd	nd	nd	nd	nd	nd	nd	nd	nd	nd	nd	nd	nd	nd	nd	nd	nd	nd	nd

Table S5. Class level community composition (%) of seawater bacterial communities. Abbreviations: nd—not detected, tr—trace abundance (<0.1%).

	Percent Composition in Seawater Samples						
**Class**	**W1**	**W2**	**W13**	**W15**	**W17**	**W18**	**W26**
Acidobacteria	tr	nd	tr	nd	tr	nd	nd
Actinobacteria	9.5	0.1	tr	0.4	0.3	4.2	tr
Alphaproteobacteria	17.6	2.8	3.1	3.2	4.6	18.1	5.0
Bacilli	6.2	nd	nd	tr	0.2	0.3	tr
Bacteroidia	nd	nd	nd	nd	nd	tr	nd
Betaproteobacteria	2.8	tr	0.1	0.2	tr	tr	0.2
Chlamydiae	tr	nd	nd	0.1	tr	nd	nd
Clostridia	5.1	tr	tr	nd	tr	tr	nd
Cyanobacteria	15.4	29.8	31.4	31.0	35.8	22.6	32.2
Deinococci	1.1	nd	tr	nd	nd	nd	tr
Deltaproteobacteria	tr	tr	nd	0.1	0.2	0.4	0.1
Epsilonproteobacteria	nd	nd	tr	nd	nd	nd	nd
Epsilonproteobacteria	nd	nd	nd	nd	nd	nd	nd
Erysipelotrichia	nd	nd	nd	nd	nd	nd	nd
Flavobacteria	5.8	2.5	4.3	6.7	5.4	3.3	7.0
Fusobacteria	nd	tr	tr	nd	tr	nd	tr
Gammaproteobacteria	5.7	6.1	6.6	5.8	5.8	10.7	6.9
Gemmatimonadetes	nd	nd	nd	nd	nd	nd	nd
Lentisphaeria	tr	nd	nd	nd	nd	nd	nd
Mollicutes	nd	nd	nd	nd	nd	nd	nd
Negativicutes	nd	nd	nd	nd	nd	nd	nd
Opitutae	tr	1.5	2.6	1.3	0.9	0.8	1.8
Planctomycetacia	nd	tr	tr	tr	0.1	0.2	0.1
Sphingobacteria	1.1	0.1	0.4	0.5	0.4	0.8	0.5
Spirochaetes	nd	nd	nd	nd	nd	nd	nd
Verrucomicrobiae	nd	nd	tr	tr	tr	0.1	tr
**TM7 *incertae sedis***	nd	nd	nd	nd	tr	tr	nd
**Unclassified Bacteria**	20.8	40.1	31.9	32.8	29.6	23.2	28.1
**Unclassified *Bacteriodetes***	0.3	1.3	tr	0.3	0.7	3.7	0.7
**Unclassified *Chloroflexi***	nd	tr	nd	nd	nd	nd	nd
**Unclassified *Cyanobacteria***	nd	nd	tr	nd	tr	nd	0.1
**Unclassified *Firmicutes***	tr	tr	nd	nd	nd	nd	nd
**Unclassified *Planctomycetes***	nd	nd	nd	nd	nd	nd	nd
**Unclassified *Proteobacteria***	8.1	15.5	18.9	17.1	15.8	11.4	17.1
**Unclassified *Verrucomicrobia***	nd	nd	nd	nd	tr	nd	nd
***Verrucomicrobia* Subdivision3**	nd	nd	nd	nd	tr	nd	nd

Table S6. Taxa contributing to dissimilarity between seawater and *A. elisabethae* bacterial communities in SIMPER analyses.

Data Set	Taxa	Contribution (%)
V1/V2	*Alphaproteobacteria*	28.1
V1/V2	*Flavobacteria*	20.6
V1/V2	unclassified Bacteria	20.2
V1/V2	*Cyanobacteria*	15.7
V4	*Gammaproteobacteria*	25.6
V4	*Cyanobacteria*	25.0
V4	unclassified Bacteria	16.8
V4	unclassified *Proteobacteria*	12.7
